# The Features of Vessel Densities and Hemorheological Parameters in Patients With Central Retinal Vein Occlusion: A Prospective Randomized Case-Control Study

**DOI:** 10.1155/joph/6634841

**Published:** 2025-10-07

**Authors:** Xuan Li, Shuyue Huang, Ziyang Chen, Hang Yuan, Like Xie, Xiaofeng Hao

**Affiliations:** ^1^Department of Ophthalmology, Eye Hospital China Academy of Chinese Medical Sciences, Beijing 100040, China; ^2^Department of Endocrinology, Capital Center For Children's Health, Capital Medical University, Beijing 100020, China; ^3^Fujian Key Laboratory of Propagated Sensation along Meridian, Fujian Academy of Chinese Medical Sciences, Fuzhou 350003, China

**Keywords:** central retinal vein occlusion, hemorheology, optic nerve, optical coherence tomography angiography, vessel density

## Abstract

**Background:** This study analyzed changes in the vessel densities (VDs) and hemorheological parameters of patients with central retinal vein occlusion (CRVO).

**Methods:** A prospective randomized case-control study was conducted, which included 80 CRVO patients (the study group) and 80 participants with normal fundus (the normal control group). Best-corrected visual acuity (BCVA), optic disc and macular VD, and other structural parameters (C/D ratio, RNFL thickness, etc.) were measured with optical coherence tomography angiography. Hemorheological parameters, including whole blood viscosity at low shear rate (LSR-WBV; 5/s) and high shear rate (HSR-WBV; 200/s), and erythrocyte aggregation index (AI), were also measured.

**Results:** LSR-WBV, HSR-WBV, and AI were significantly higher in CRVO patients (9.009 ± 1.595 mPa·s, 4.981 ± 0.617 mPa·s, and 3.405 ± 1.679) than in participants with normal fundus (8.409 ± 1.110 mPa·s, 4.523 ± 0.597 mPa·s, and 2.880 ± 1.517) (all *p* < 0.05). CRVO eyes had significantly lower visual acuity, smaller C/D and cup volume, thicker peripapillary RNFL, and lower optic disc and macular VD than unaffected eyes and normal eyes (all *p* < 0.05). VD inside the optic disc and deep capillary plexus in unaffected eyes was markedly decreased when compared with that in normal eyes (all *p* < 0.05). BCVA in CRVO eyes was particularly correlated with VD inside disc (*p*=0.001 and *r* = −0.391).

**Conclusion:** CRVO patients presented with more serious thrombophilia and higher hemorheological parameters, and the blood perfusion was significantly decreased in the optic disc and macula of CRVO eyes and was partially reduced in unaffected eyes. Moreover, optic disc blood perfusion exerted greater impacts on visual acuity, and inside-disc VD might be the greatest risk factor.

## 1. Introduction

Central retinal vein occlusion (CRVO) is a common retinal vascular disease, usually leading to impaired venous outflow, retinal ischemia, and severe visual loss. The prevalence of CRVO among people aged 30−89 years was 0.08–0.13%, which has been constantly increasing in the last 7 years [1]. It particularly occurs among elderly individuals with systemic vascular risk factors such as hypertension, diabetes mellitus, and hyperlipidemia. Despite advances in diagnostic and therapeutic approaches, the pathophysiological mechanisms underlying CRVO remain incompletely understood.

Emerging evidence highlighted the importance of retinal vessel density (VD) as a critical parameter reflecting microvascular and perfusion status alternation in retinal diseases. Since the optic disc is the primary site of retinal vein occlusion, optic disc blood perfusion plays an essential role in the pathogenesis of CRVO. Therefore, the quantitative analysis of optic disc blood perfusion has garnered growing attention [2]. Optical coherence tomography angiography (OCTA) is a noninvasive and rapid imaging technique that generates in vivo cross-sectional images of dynamic microvessels in the retina, providing valuable information on the VD of the optic disc in CRVO eyes [3]. Hemorheological abnormality, described as a “hypercoagulable state,” is another characteristic of CRVO, including increased blood viscosity, altered erythrocyte deformability, and abnormal platelet aggregation [4]. Hemorheological changes may relate to the progression of CRVO by promoting thrombus formation and vascular occlusion.

In the present study, we aim to investigate the underlying causes of CRVO through deep observation of blood perfusion following CRVO from two aspects of VD and hemorheological changes, thus revealing the pathogenesis of CRVO and the risk factors of CRVO-related visual impairment.

## 2. Methods

### 2.1. Study Design and Participants

This prospective randomized case-control study was performed from May 2021 to May 2023, included 80 treatment-naïve patients diagnosed with CRVO within 30 days of onset, and 80 age- and gender-matched healthy participants without any fundus abnormalities. This study was approved by the Ethics Committee of the Eye Hospital of China Academy of Chinese Medical Science (no. YKEC-KT-2022-024-P002), and informed consent was obtained from all the participants in the study.

### 2.2. Inclusion and Exclusion Criteria

The diagnostic criteria of CRVO were referred to RETINA (Fifth Edition) [5]: sudden vision loss or visual field defect; retinal vein tortuosity and dilatation and typical superficial retinal hemorrhage; and fluorescein angiography (FFA) showed delayed retinal venous filling time, vascular leakage, tortuous dilation of capillaries, and sometimes optic disc edema.

Inclusion criteria were as follows: (1) a diagnosis of CRVO as per RETINA (5th edition) diagnostic guidelines [5]; (2) no previous treatment for CRVO; and (3) diagnosis within 90 days of symptom onset. Exclusion criteria included the presence of other ocular diseases (e.g., diabetic retinopathy, retinal artery occlusion, uveitis, glaucoma, or ocular trauma) and systemic diseases affecting hemorheological parameters, such as diabetes mellitus, hypertension, hyperlipidemia, anemia, polycythemia vera, thrombocytosis, leukocytosis, autoimmune disorders, and active infections.

### 2.3. Ophthalmologic Examinations

The VD of the optic disc, including VD in the whole optic disc, inside-disc VD, and peripapillary VD, was measured with OCTA (RTVue XR; OPTOVUE Inc., Fremont, California, USA) in the Angio Retina mode (optic disc: 4.5 × 4.5 mm). Peripapillary retinal nerve fiber layer (RNFL) thickness, vertical cup/disc (C/D), optic disc area, and cup volume were also measured. The VD of the superficial capillary plexus (SCP) and deep capillary plexus (DCP), as well as central macular thickness (CMT), was measured in the Angio Retina mode (macula: 3 × 3 mm).

The best-corrected visual acuity (BCVA) was examined with Early Treatment Diabetic Retinopathy Study letter scores and converted to logarithm of the minimum angle of resolution (logMAR) values. Intraocular pressure (IOP) was also examined. Additionally, slit-lamp examination, fundus photography, and FFA (TRC-50DX; Heidelberg Inc., Heidelberg, Germany) were performed.

### 2.4. Hemorheological Examinations

To assess the hemorheological features in CRVO, 5 mL of EDTA-anticoagulated venous blood was collected and analyzed by an automatic hemorheology analyzer (South900, Chongqing Nanfang numerical control equipment Co., Ltd., China). Whole blood viscosity at low shear rate (LSR-WBV; 5/s) and high shear rate (HSR-WBV, 200/s), erythrocyte aggregation index (AI), plasma viscosity (PV), hematocrit (HCT), erythrocyte rigidity index (IR), and deformability index (DI) were measured according to the manufacturer's instructions. All measurements were performed by trained laboratory technicians blinded to the clinical data.

### 2.5. Statistical Analysis

Statistical analysis was conducted with IBM SPSS 23.0. The Shapiro–Wilk test was used to assess the normality of continuous variables. Data following a normal distribution were presented as the mean ± standard deviation and compared using independent sample *t*-tests. Data not conforming to normal distribution were expressed as the median (interquartile range) and compared using the Mann–Whitney *U* test. The Spearman correlation coefficient was used for correlation analysis. Differences with *p* < 0.05 were considered statistically significant.

## 3. Results

### 3.1. General Information of Participants

Eighty CRVO patients (80 CRVO eyes) were included as the study group (age of 35–88 years, with a mean age of 58.9 ± 12.4 years), including 42 males and 38 females. Eighty participants with normal fundus were enrolled as the normal control group.

### 3.2. Visual Acuity of Participants

BCVA was 0.10–3.00 logMAR (mean: 1.225 ± 0.684) in CRVO eyes, −0.10−0.20 logMAR (mean: 0.021 ± 0.071) in the unaffected eyes of the study group, and −0.20−0.20 logMAR (mean: 0.008 ± 0.082) in eyes of healthy subjects. BCVA was significantly lower in CRVO eyes than in unaffected eyes (*p*=0.001) and normal eyes (*p*=0.001).

### 3.3. Hemorheological Parameters of Participants

Compared with participants with normal fundus, CRVO patients exhibited increases in whole blood viscosity at low shear rate (LSR-WBV; 5/s) and high shear rate (HSR-WBV; 200/s), PV, HCT, erythrocyte AI, erythrocyte rigidity index (IR), and erythrocyte DI, among which increases in LSR-WBV, HSR-WBV, and AI were statistically significant (*p* < 0.05), as shown in Table 1.

### 3.4. Structural Features and VD Changes of the Optic Disc

Vertical C/D and optic cup volume were markedly smaller and peripapillary RNFL was substantially thicker in CRVO eyes than in unaffected and normal eyes (*p* < 0.05). Unaffected eyes had insignificantly smaller vertical C/D and optic cup volume than normal eyes (*p* > 0.05).

Optic disc VD in CRVO eyes was significantly lower than that in unaffected and normal eyes (*p* < 0.05). Compared with normal eyes, inside-disc VD was prominently reduced in unaffected eyes (*p* < 0.05), as shown in Table 2.

CMT was significantly thicker and SCP-VD and DCP-VD were markedly lower in CRVO eyes than in unaffected and normal eyes (*p* < 0.05). Compared with normal eyes, unaffected eyes displayed lower DCP-VD (*p* < 0.05), as shown in Table 3.

### 3.5. Risk Factors of Visual Impairment in CRVO Eyes

**Figure 1:**
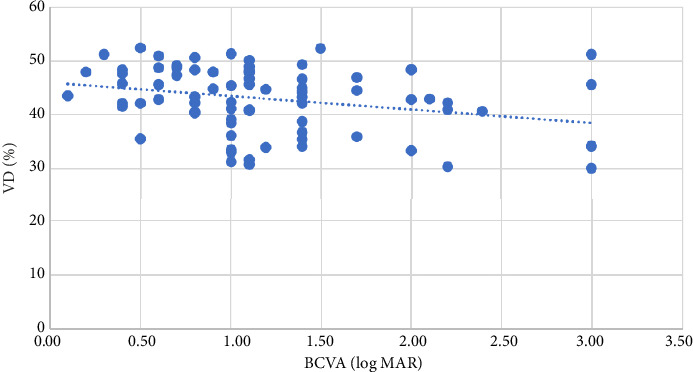
The relationship between whole disc VD and BCVA (logMAR).

**Figure 2:**
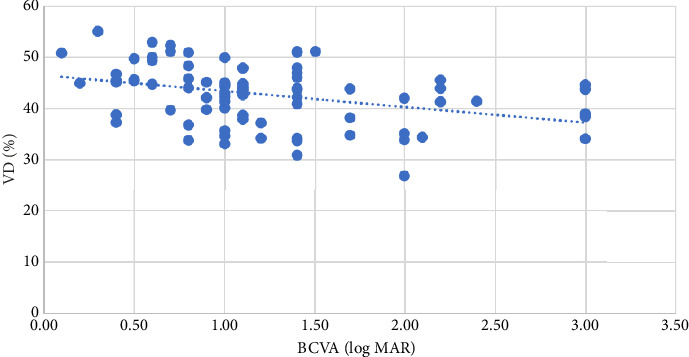
The relationship between inside-disc VD and BCVA (logMAR).

**Figure 3:**
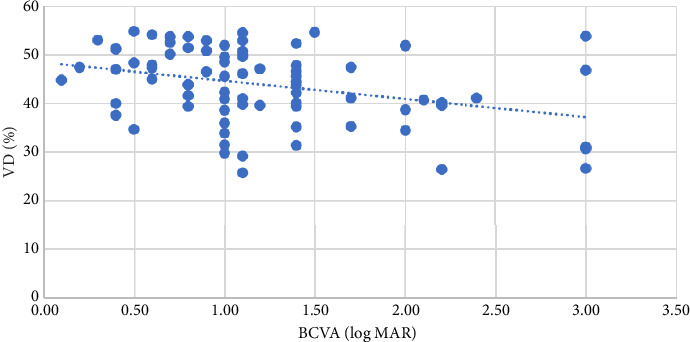
The relationship between peripapillary VD and BCVA (logMAR).

BCVA (logMAR) was correlated with VD in the whole disc (*p*=0.015 and *r* = −0.271 at the level of 0.05), inside-disc VD (*p*=0.001 and *r* = −0.391 at the level of 0.01), and peripapillary VD (*p*=0.005 and *r* = −0.309 at the level of 0.01), with the strongest correlation between BCVA and inside-disc VD (Table 4), as shown in Figures 1, 2, and 3.

## 4. Discussion

### 4.1. Hemorheological Disorders

Despite uncertainty about the exact pathogenesis of CRVO, thrombosis is still considered the main cause of CRVO. Forty years ago, Green et al. autopsied 29 cadaver eyes with CRVO and found the presence of fresh or recanalized thrombi in the lamina cribrosa of each eye [6], implicating that thrombosis is the crucial step in the onset of CRVO, manifested as alterations in hemorheological parameters. Luo Wenling tested the hemorheological parameters of 25 RVO patients and observed that HCT and WBV were within the normal range but markedly higher than those in healthy individuals [7]. Similarly, the current study unveiled that all hemorheological parameters of CRVO patients were higher than those of participants with normal fundus, with prominent differences in LSR-WBV, HSR-WBV, and AI between the two groups, indicating thicker and more easily coagulated blood in CRVO patients, a characteristic mainly associated with erythrocyte aggregation.

### 4.2. Optic Disc Structure and VD Changes in CRVO Eyes

A prior study elucidated that the diameter of retinal veins and arteries passing through the optic disc was significantly positively correlated with the size of the optic disc, particularly in CRVO eyes [8]. The research by Longo et al. revealed that the optic disc size decreased in 25% of CRVO patients and speculated that increased pressure in the closed space of the scleral outlet at the lamina might be the main pathogenesis of CRVO [9]. In our study, the optic disc area was larger in CRVO eyes than that in unaffected and normal eyes, which might be a result of optic disc edema. Furthermore, two eyes of CRVO patients, particularly CRVO eyes, had smaller C/D and cup volume than the eyes of participants with normal fundus, the same as the findings of Lei, who defined the optic disc with C/D of less than or equal to 0.2 as a “high-risk disc” of CRVO [10].

As a novel examination method, OCTA has the advantages of being quick, noninvasive, and accurate. This method can clearly visualize the retinal capillary plexus in different layers without interference from retinal hemorrhage or vascular leakage and, therefore, can quantitatively characterize blood perfusion as presented by VD [11]. In the present study, OCTA was utilized to quantitatively analyze the blood perfusion of the optic disc and macula. The results displayed that optic disc and macular VD in CRVO eyes were remarkably lower than those in unaffected and normal eyes, consistent with the pathological mechanism of CRVO reported in a previous study [12]. Our data also exhibited that optic disc VD and DCP-VD were significantly decreased in unaffected eyes when compared with normal eyes. Wang et al. also observed a similar phenomenon and suggested that underlying vascular occlusion might occur in the unaffected eye of patients and that changes in blood perfusion might precede the onset of CRVO [13].

### 4.3. Risk Factors of Visual Impairment in CRVO Eyes

As the most frequent complication of CRVO, macular edema is defined as CMT ≥ 250 μm and is believed as the predominant cause of visual impairment after CRVO [14]. Our study revealed that CMT in CRVO eyes (mean: 525 μm) was substantially elevated as compared with that in unaffected and normal eyes, supporting the diagnosis of macular edema. However, no direct correlation between logMAR of BCVA and CMT in CRVO eyes was observed in our study, whilst a close correlation was found between BCVA and blood perfusion of the optic disc. Inside-disc VD had the strongest correlation with BCVA, illustrating inside disc VD as the most significant risk factor of visual impairment in CRVO eyes, which was first proposed in this study.

## 5. Limitations

This study had a limitation. The onset times of patients in this study ranged from several hours to 80 days. Hence, these patients were in different stages of the disease. Due to the limited sample size, the correlation between the blood perfusion of the optic disc and the stage of the disease was not further analyzed, calling for follow-up research. Accordingly, studies with larger sample sizes are warranted for further studies.

## 6. Conclusions

Conclusively, CRVO patients presented with obviously higher WBV and AI, indicating that thrombophilia was more serious in CRVO patients and that erythrocyte aggregation might assume a key role in the progression of CRVO. Optic disc and macular VD were significantly reduced in CRVO eyes and even partially decreased in unaffected eyes, underscoring that decreased VD may be a predictor of CRVO. Although macular edema was the primary cause of visual impairment in CRVO eyes, blood perfusion of the optic disc had a stronger impact on visual acuity, and inside-disc VD might be the most important risk factor for visual impairment in CRVO eyes.

## Figures and Tables

**Table 1:** Comparisons of hemorheological parameters between CRVO patients and participants with normal fundus.

	*N*	LSR-WBV (5/s)	HSR-WBV (200/s)	PV (mPa·s)	HCT (L/L)	AI	IR	DI
		(mPa·s)	(mPa·s)					
CRVO patients	80	9.009 ± 1.595	4.981 ± 0.617	1.531 ± 0.263	0.428 ± 0.035	3.405 ± 1.679	5.622 ± 1.337	0.983 ± 0.694
Participants with normal fundus	80	8.409 ± 1.110	4.523 ± 0.597	1.448 ± 0.177	0.412 ± 0.057	2.880 ± 1.517	5.190 ± 1.374	0.895 ± 0.135
*p*		**0.008**	**0.000**	0.072	0.059	**0.026**	0.094	0.947

*Note:* LSR-WBV, whole blood viscosity at low shear rate; HSR-WBV, whole blood viscosity at high shear rate; HCT, hematocrit; AI, erythrocyte aggregation index; IR, erythrocyte rigidity index. Bold values stand for statistically significant values (*p* < 0.05).

Abbreviations: DI, deformability index; PV, plasma viscosity.

**Table 2:** Comparisons of parameters of optic disc among CRVO, unaffected, and normal eyes.

	*N*	Vertical C/D	Optic disc area (mm^2^)	Optic cup volume (mm^3^)	RNFL thickness (μm)	Whole optic disc (%)	Inside disc (%)	Peripapillary (%)
CRVO eyes	80	0.167 ± 0.219	2.160 ± 0.586	0.0310 ± 0.0684	159.1 ± 48.9	42.88 ± 5.95	42.59 ± 5.82	43.72 ± 7.66
Unaffected eyes	80	0.251 ± 0.203	2.072 ± 0.507	0.0441 ± 0.0577	113.9 ± 12.3	49.87 ± 2.70	49.91 ± 5.11	52.48 ± 3.26
Normal eyes	80	0.277 ± 0.143	2.118 ± 0.382	0.0525 ± 0.0654	111.8 ± 13.6	49.22 ± 2.63	51.52 ± 4.44	52.22 ± 2.87
*P*_1_		**0.000**	0.113	**0.000**	**0.000**	**0.000**	**0.000**	**0.000**
*P*_2_		**0.000**	0.520	**0.000**	**0.000**	**0.000**	**0.000**	**0.000**
*P*_3_		0.412	0.270	0.155	0.157	0.101	**0.037**	0.459

*Note:P*_1_ represented comparisons between CRVO and unaffected eyes. *P*_2_ represented comparisons between CRVO and normal eyes. *P*_3_ represented comparisons between unaffected and normal eyes. Structural characteristics and vascular density changes of macula. Bold values stand for statistically significant values (*p* < 0.05).

**Table 3:** Comparisons of central foveal thickness and VD in superficial capillary plexus and deep capillary plexus among CRVO, unaffected, and normal eyes.

	*N*	CMT	SCP-VD (%)	DCP-VD (%)
		(μm)		
CRVO eyes	80	526.4 ± 208.4	40.20 ± 5.64	39.55 ± 6.55
Unaffected eyes	80	236.4 ± 12.9	46.27 ± 3.40	49.59 ± 3.78
Normal eyes	80	234.0 ± 16.8	46.56 ± 2.61	50.81 ± 2.36
*P*_1_		**0.000**	**0.000**	**0.000**
*P*_2_		**0.000**	**0.000**	**0.000**
*P*_3_		0.379	0.484	**0.021**

*Note:P*_1_ represented comparisons between CRVO and unaffected eyes. *P*_2_ represented comparisons between CRVO and normal eyes. *P*_3_ represented comparisons between unaffected and normal eyes. Bold values stand for statistically significant values (*p* < 0.05).

**Table 4:** Risk factors of BCVA in CRVO.

Parameters	*r*_*s*_	*p*
Vertical C/D	0.000	0.287
Optic disc area (mm^2^)	−0.103	0.364
Optic cup volume (mm^3^)	−0.154	0.172
RNFL thickness (μm)	−0.156	0.168
Whole optic disc (%)	−0.271^∗^	0.015
Inside disc (%)	−0.391^∗∗^	0.000
Peripapillary (%)	−0.309^∗∗^	0.005
SCP-VD (%)	−0.176	0.118
DCP-VD (%)	−0.061	0.590
CMT (μm)	−0.073	0.522
LSR-WBV (5/s)(mPa·s)	0.125	0.269
HSR-WBV (200/s)(mPa·s)	0.100	0.376
PV (mPa·s)	0.196	0.081
HCT (L/L)	0.089	0.434
AI	0.116	0.306
IR	−0.110	0.329
DI	−0.108	0.340

^∗^Stands for statistical significance at the 0.05 level (*p* < 0.05).

^∗∗^Stands for statistical significance at the 0.01 level (*p* < 0.01).

## Data Availability

The data that support the findings of this study are available from the corresponding authors upon reasonable request.

## Nomenclature

VD: Vessel densities
CRVO: Central retinal vein occlusion
C/D: Cup/disc
RNFL: Retinal nerve fiber layer
CMT: Central macular thickness
PV: Plasma viscosity
HCT: Hematocrit
AI: Aggregation index
DI: Deformability index
BCVA: Best-corrected visual acuity
OCTA: Optical coherence tomography angiography
SCP: Superficial capillary plexus
DCP: Deep capillary plexus
CMT: Central macular thickness
IOP: Intraocular pressure
logMAR: Logarithm of the minimum angle of resolution
